# Regular, moderate intensity maternal exercise reduces birth weight but increases the risk of later childhood adiposity

**DOI:** 10.1186/1687-9856-2015-S1-O43

**Published:** 2015-04-28

**Authors:** Valentina Chiavaroli, Sarah Hopkins, Janene Biggs, José Derraik, Raquel Olmedo Rodrigues, Wayne Cutfield, Paul Hofman

**Affiliations:** 1 Liggins Institute, University of Auckland, Auckland, New Zealand

## Aims

We have previously randomised primiparous mothers to either an exercise regime or normal activity between 20 and 36 weeks gestation. In this study we aimed to assess the long-term effects of exercise during pregnancy on growth parameters and body composition in the offspring over their first 6-8 years of life.

## Methods

Of the initial 84 women and their offspring who participated in the RCT, follow-up data were available on 46 mothers (26 exercisers, 20 controls) and 46 children. At each follow-up visit (6 months, 1 year, 2 years, 4 years, 6-8 years) clinical assessment included measurement of mothers’ and children’s heights, weights, BMI, and waist circumference, as well as blood pressure. Body composition was assessed in both mothers and children by whole-body dual-energy X-ray absorptiometry (DXA) scans at 4-year and 7-year follow-up visits.

## Results

There were no differences in anthropometry between exercise and control children in the first 2 years of life. In addition, at age 4 years there were no differences in height, BMI, percentage body fat, or waist circumference between the two groups. At a mean age of ~7.5 years, exercise and control children showed similar weight, height, BMI, and waist circumference, but the exercise group had more body fat (17.5 vs 16.0%, P=0.02) than controls. Over the course of follow-up there were no observed differences in anthropometry between exercise and control mothers.

## Conclusion

While no long-term benefits of maternal exercise in the first pregnancy were noted in mothers, children exposed to maternal exercise during intrauterine life appear prone to greater fat mass accumulation in mid-childhood. Larger studies are required to confirm this important observation as exercise in pregnancy is widely recommended by obstetricians.

